# Localized massive staphylococcal pericardial abscess with atypical presentation

**DOI:** 10.1016/j.amsu.2022.103502

**Published:** 2022-03-12

**Authors:** Abdinafic Mohamud Hussein, Erhan Renan Ucaroglu, Abdirahman Mohamed Hassan Dirie, Mohamed Farah Yusuf, Abdirahman Abdulkadir Osman

**Affiliations:** a Department of Cardiovascular Surgery, Somalia Turkey Recep Tayyip Erdoğan Training and Research Hospital, Mogadishu, Somalia; b Department of Pulmonology, Somalia Turkey Recep Tayyip Erdoğan Training and Research Hospital, Mogadishu, Somalia; c Department of Emergency Medicine, Somalia Turkey Recep Tayyip Erdoğan Training and Research Hospital, Mogadishu, Somalia; d Department of Internal Medicine, Somalia Turkey Recep Tayyip Erdoğan Training and Research Hospital, Mogadishu, Somalia

**Keywords:** Localized pericardial abscess, Pericardial abscess, Staphylococcus aureus, Renal failure

## Abstract

**Introduction and importance:**

Localized staphylococcal pericardial abscess (PA) is extremely rare and highly mortal complication of Staphylococcus aureus bacteremia with only a few reported cases in English-language medical literature. Clinical manifestations are fulminant, and early management is necessary.

**Case presentation:**

Here we report a case of end stage renal disease (ESRD) with isolated localized massive staphylococcal PA and had masked signs and symptoms of pericardial staphylococcal infection. Contrast-enhanced computed tomography scan suggested anterior massive localized pericardial cyst like image. Surgical drainage of the abscess with localized pericardiectomy conjugated with antibiotic therapy led to a successful management.

**Clinical discussion:**

Staphylococcus aureus is the leading cause of hemodialysis catheter-related bloodstream infections, contributing 33–80% of the organisms cultured from blood samples. Nature of staphylococcal pericardial infection is aggressive and life threatening with generalized involvement of the pericardium although our patient presented with masked signs and symptoms.

**Conclusion:**

CT image of localized pericardial lesion with masked signs and symptoms does not exclude the presence of live threating pericardial infection, especially in immunocompromised patients.

## Highlights

• Localized staphylococcal pericardial abscess is an extremely rare condition with only a few reported cases in English-language medical literature.
• CT images of localized pericardial abscess mimics with that of pericardial cysts, and can lead to misdiagnosis; this is particularly true when there is an indolent presentation of the condition caused by immunosuppression.
• Successful management can be achieved through surgical drainage of the abscess antibiotic therapy according to the bacterial sensitivity.

## 1 Introduction

Pericardial abscess is considered as an extremely rare complication of bacteremia and delayed diagnosis can be very serious [1]. Risk factors include diabetes mellitus, other active infection (eg, endocarditis or pneumonia), immunosuppression, and having a preexisting pericardial effusion [2]. PA occurs in patients with immunosuppression as secondary complication, although since the advent of antibiotic therapy, the incidence of PA has decreased [3]. It usually presents as generalized involvement of the pericardium, but here we report a case with localized massive staphylococcal pericardial abscess [4,5]. The diagnosis and management of pericardial diseases in General remain challenging because of the vast spectrum of manifestations of the condition [6].

## 2 Case report

A 45-years old woman was referred to the cardiovascular surgery department of our hospital complaining about dyspnea, orthopnea, cough, chest pain and peripheral edema for three weeks. She had a history of chronic renal failure and has been on dialysis for 6 months prior to her hospitalization. Patient underwent 2 times temporary hemodialysis catheterizations due to failure of her radio-cephalic fistula caused by hypotension. First hemodialysis catheter was removed 5 weeks before admission due to hemodialysis catheter infection, with blood culture being positive for Methicillin-resistant Staphylococcus aureus(MRSA). She responded well to medical treatment. She also had a history of hypertension for 2 years but had no history of previous surgery.

On examination, her vital signs were as follows: pulse 111beats/min, blood pressure 70/40 mmHg, afebrile with body temperature 37.0 °C, respiratory rate 20/min and oxygen saturation on room air was 90%. On auscultation, there were distant heart sounds, elevated jugular venous pressure and bilateral lower limb edema.

**Fig. 1:**
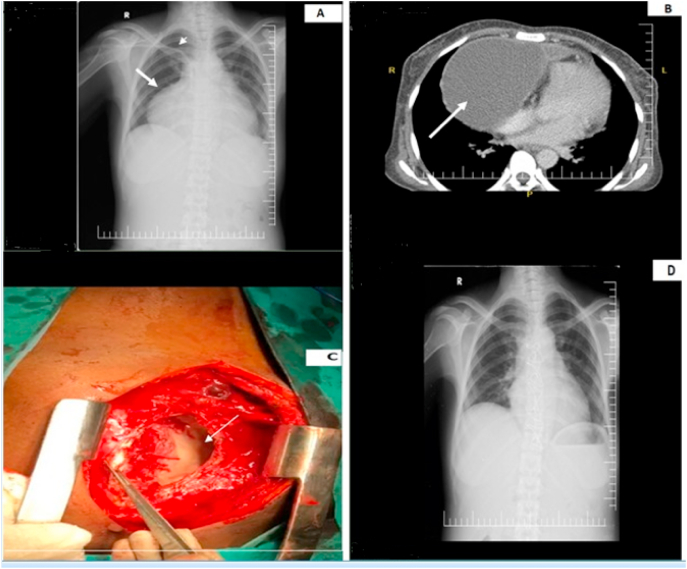
(A) Chest x-ray on admission showing protrusion of the right heart border and enlarged cardiac silhouette. There is also right subclavian hemodialysis catheter. (B) Contrast-enhanced computed tomography scan (CT-scan) of the chest, demonstrating a localized massive pericardial abscess on the right anterior side of the heart. (C) Intraoperative image of localized pericardial abscess (arrow) after a median sternotomy. (D) Chest x-ray on five weeks after operation showing normal cardiac size and recovery.

Laboratory findings showed leukocytosis 11.0 × 109/L with neutrophils predominance 75%, hemoglobin 9.2g/dL, elevated high sensitive C-reactive protein level of 97 mg/dl, creatinine 9.8 mg/dl, blood urea 180 mg/dl. Electrocardiogram showed sinus tachycardia with low voltage complexes and chest X-ray showed cardiomegaly (Fig. 1. A). Transthoracic echocardiography suggested massive anterior pericardial collection with right ventricular diastolic collapse and Contrast-enhanced computed tomography scan suggested anterior massive localized pericardial collection just similar to that of pericardial cyst (Fig. 1. B).

Considering the possibility of massive pericardial cyst, urgent surgical intervention was considered. The operation was performed by cardiovascular surgery specialist. A median sternotomy was made, followed by opening of the pericardium; a localized abscess of around 600ml was exposed and drained from the anterior aspect of the heart, and partial pericardiectomy was performed (Fig. 1. C). Culture of the pus yielded growth of MRSA and pathological examination of the pericardial specimen showed non-specific inflammation. Linezolid was started according to bacterial sensitivity and patient stayed at the hospital for 10 days. She made a good postoperative recovery. She completed additional 2 weeks of antibiotic treatment. After five weeks, she came for follow up and chest x-ray showed complete recovery (Fig. 1. D). This case has been reported in line with the SCARE 2020 criteria [7].

## 3 Discussion

Localized staphylococcal pericardial abscess is an extremely rare and highly mortal complication of Staphylococcus aureus bacteremia with only a few reported cases in English-language medical literature [2,5]. pericardial infections can result from hematogenous spread, direct extension from pneumonia, empyema, chest trauma, or postoperative mediastinitis [1]. according to the present case there is a high possibility that pericardial abscess may resulted from hematagenous spread of staphylococcus aureus from staphylococcus infected hemodialysis catheter since blood culture showed growth of staphylococcus aureus 6 weeks prior to her admission, although the mechanism by which Staphylococcus aureus forms a local pericardial abscess is unknown [5]. Staphylococcus aureus is the leading cause of hemodialysis catheter-related bloodstream infections, contributing 33–80% of the organisms cultured from blood samples [8].

Nature of staphylococcal pericardial infection is aggressive and life threatening and it was found that the progression is very rapid, and half of all cases are diagnosed on autopsy [9]. The case presented here shows atypical presentation of the disease since there was no fever and there was a subtle increase of the signs and symptoms for up to three weeks before her hospitalization. The indolent cause of the presentation could be due to the chronic renal failure induced immunosuppression [10].

Our preoperative diagnosis of this patient was massive anterior pericardial cyst that is functionally compromising the right side of the heart due to the CT image and masked signs and symptoms of pericardial staphylococcal infection. Unusual presentations for abscess formation are not uncommon [11,12]. Therefore, absence of aggressive sign and symptoms of infection and presence of localized pericardial lesion could not exclude the presence of pericardial abscess.

Regarding to the management of bacterial pericarditis, aggressive antibiotic therapy according to the bacterial sensitivity has to be conjugated with surgical or percutaneous drainage of the abscess. Surgical drainage is preferred as it permits pericardiectomy in case of constriction [8]. As reported previously, Linezolid is associated with high frequency of linezolid-induced thrombocytopenia and anemia among patients with ESRD but our case had not experienced such complications [13].

Although successful management was achieved in our patient, mortality rate remains high of 45% even in those who received appropriate treatment [8].

## 4 Conclusion

Localized staphylococcal pericardial abscess is an extremely rare and highly mortal complication of Staphylococcus aureus bacteremia with only a few reported cases in English-language medical literature. CT images of localized pericardial abscess can be confused with that of pericardial cysts. Patients can present with atypical signs and symptoms of pericardial staphylococcal infection. Successful management could be achieved through aggressive antibiotic therapy according to the bacterial sensitivity conjugated with surgical drainage of the abscess.

## Ethical approval

N/A.

## Funding source

There is no funding source for this study.

## Author contribution

All authors contributed toward writing, analysis, drafting, and revising of the paper.

## Registration of research studies

Name of the registry: Not applicable.

Unique Identifying number or registration ID: Not applicable.

Hyperlink to your specific registration (must be publicly accessible and will be checked): Not applicable.

## Guarantor

Abdinafic Mohamud Hussein.

## Consent to participate

Written informed consent was obtained from the patient for publication of this case report and accompanying images. A copy of the written consent is available for review by the Editor-in-Chief of this journal on request.

## Provenance and peer review

Not commissioned, externally peer reviewed.

## Declaration of competing interest

The authors declare that there is no competing interest related to the study, authors, other individuals, or organizations.

## References

[1] Pérez-Cardona J., Salgado V., Medina-Ruiz A., Quilinchini J.M.A. (2008).

[2] Wright N.R., Pfahl K.W., Bush C.A. (2016). Purulent pericarditis and abscessed myocardium with acute myocardial infarction. Am. J. Med..

[3] Nakao Y., Higaki T., Nakama Y., Morito T., Suenari K., Nishioka K. (2019). Primary pericardial abscess caused by Staphylococcus aureus infection without a predisposing condition. J. Cardiol. Cases..

[4] Hussein A.M., Korkmaz U.T.K., Yapici K., Ali A.A., Kizilay M. (2021). Successfully managed case of cardiac tamponade due to tuberculous pericardial effusion: a case study. Iran. Hear. J..

[5] van de Donk N.W.C.J., Mijer R.C.A., America Y.G.C.J., Cramer M.J. (2010). Staphylococcus aureus pericardial abscess presenting as a localized bulge of the heart contour. Interact. Cardiovasc. Thorac. Surg..

[6] Khandaker M.H., Espinosa R.E., Nishimura R.A., Sinak L.J., Hayes S.N., Melduni R.M. (2010). Pericardial disease: diagnosis and management. Mayo Clin. Proc..

[7] Agha R.A., Franchi T., Sohrabi C., Mathew G., Kerwan A., Thoma A. (2020). The SCARE 2020 guideline: updating consensus surgical CAse REport (SCARE) guidelines. Int. J. Surg..

[8] Singh N.P., Prakash A., Makhija A., Garg D., Pathania A., Agarwal S.K. (2009).

[9] Kaye A., Peters G.A., Joseph J.W., Wong M.L. (2019). Purulent bacterial pericarditis from Staphylococcus aureus. Clin. Case. Rep..

[10] Kato S., Chmielewski M., Honda H., Pecoits-Filho R., Matsuo S., Yuzawa Y. (2008). Aspects of immune dysfunction in end-stage renal disease. Clin. J. Am. Soc. Nephrol..

[11] Hasan A., Nafie K., Monazea K., Othman A., Salem A., Ismail A. (2020). A rare case of recurrent eccrine poroma underlying gluteal abscess. Int. J. Surg .Case. Rep..

[12] Srivanitchapoom C., Yata K. (2021). Clinical characteristics that predict parotid abscess: an observational cohort study. Ann. Med. Surg..

[13] Crass R.L., Cojutti P.G., Pai M.P., Pea F. (2019). Reappraisal of linezolid dosing in renal impairment to improve safety. Antimicrob. Agents Chemother..

